# Synthesis of Well-Ordered Functionalized Silicon Microwires Using Displacement Talbot Lithography for Photocatalysis

**DOI:** 10.1021/acsomega.4c03039

**Published:** 2024-04-25

**Authors:** Axl Eriksson, Anurag Kawde, Lukas Hrachowina, Sarah R. McKibbin, Qi Shi, Magnus T. Borgström, Thomas Wågberg, Tönu Pullerits, Jens Uhlig

**Affiliations:** † Chemical Physics, Department of Chemistry, Lund University, Kemicentrum Naturvetarevägen 16, Lund 223 62, Sweden; ‡ Lund Institute of Advanced Neutron and X-ray Science, Lund University, Scheelevägen 19, Lund 223 70, Sweden; § NanoLund, Department of Physics, Lund University, Professorsgatan 1, Lund 223 63, Sweden; ∥ Solid State Physics, Department of Physics, Lund University, Professorsgatan 1, Lund 223 63, Sweden; ⊥ Department of Physics, Umeå University, Linnaeus väg 20, Umeå 907 36, Sweden; # Wallenberg Initiative Materials Science for Sustainability, Department of Physics, Umeå University, Umeå 901 87, Sweden

## Abstract

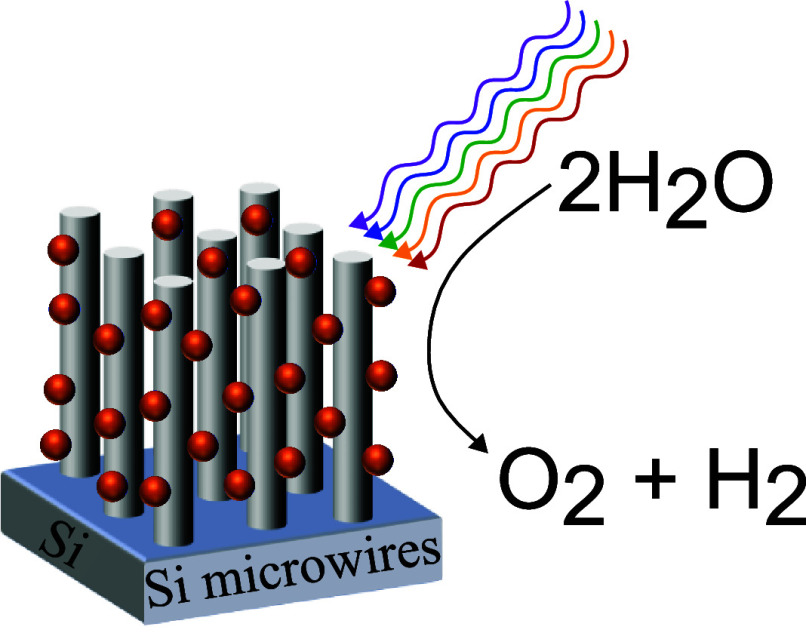

Metal-assisted chemical etching (MACE) is a cheap and scalable method that is commonly used to obtain silicon nano- or microwires but lacks spatial control. Herein, we present a synthesis method for producing vertical and highly periodic silicon microwires, using displacement Talbot lithography before wet etching with MACE. The functionalized periodic silicon microwires show 65% higher PEC performance and 2.3 mA/cm^2^ higher net photocurrent at 0 V compared to functionalized, randomly distributed microwires obtained by conventional MACE at the same potentials.

## Introduction

One important stream in the field of solar light harvesting is the work on photoelectrochemical (PEC) cells that combine light absorption with a catalytic reaction to store the generated energy in chemical bonds as in H_2_ generation from water splitting.^1^ The water splitting reaction consists of two half reactions: the oxygen evolution reaction, which results in O_2_, 4H^+^ and 4 electrons to be used in the second half reaction, the hydrogen evolution reaction, which produces H_2_. The two reactions are illustrated in eqs 1 and 2 below.^1^
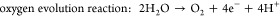
1
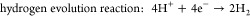
2

Ongoing research focuses on designing materials that catalyze the half reactions to reduce the overpotential.^2,3^ In a PEC cell, the light-absorbing and catalytic areas scale together, and the usage of earth-abundant elements promises easier scalability for potential future deployment. This has motivated the development of a range of cost-effective photoelectrodes whose functionality is based on materials such as Fe_2_O_3_,^4,5^ BiVO_4_,^6^ TiO_2_,^7−10^ and Si.^9,11−13^ Among these, Si is particularly interesting due to its close-to-optimum bandgap of 1.1 eV that can convert up to ∼44% of incident solar energy to H_2_, akin to the Shockley–Queisser limit, and connection to large-scale fabrication lines.^1^ A polished silicon wafer reflects a large fraction of the incident solar light due to its mirror-like appearance.^8^ This is commonly avoided by structuring the surface to reduce the reflection, which is colloquially called “black silicon”.^9^ Elongated structures additionally introduce a beneficial directionality of the charge transfer, which has been explored especially in Si nanowires.^9,12^ One of the most common and highly used methods to generate Si nanowires is metal-assisted chemical etching (MACE) of Si.^14^ The common method for MACE is to deposit metal nanoparticles via a solution process that results in a random distribution with varying densities and lacking periodicity over large area wafers. The reader is referred to relevant literature for a thorough review on the working principles of MACE.^15^ The different spacing between nanowires makes their functionalization with cocatalyst for solar catalysis challenging.^11^ To better control the distance between Si nanowires and achieve periodic patterns, classic lithography has been combined with electron beam (E-beam)^16^ and nanosphere-assisted reactive ion etching (NS-RIE).^17^ The main challenge in this lithography process, however, is to achieve spatial precision better than 10 nm across large wafer areas while maintaining the low cost and robustness of the process. Though E-beam lithography can create features below 10 nm, it comes at a considerably high cost. NS-RIE can be cost-effective, but the spatial precision and higher uniformity of the deposition of colloidal spheres (due to self-assembly) are challenging on a wafer scale. In this paper, we report on a fabrication method based on the highly cost-effective displacement Talbot lithography (DTL), which can create well-ordered light patterns on a wafer scale as the basis for well-ordered 3D nanostructures.^18−20^ In DTL, multiple-wave interference results in a self-image on the photoresists (spin-coated on the substrate) below a grating mask illuminated by a coherent light source.^21^ Different patterns and feature shapes can be obtained by varying the phase mask (grating) or illuminating multiple times by displacing the wafer after each light exposure, which is done by use of double displacement Talbot lithography (D2TL) as reported by Shields et al.^19^ The scale and resolution of the interference pattern is defined by the Talbot distance^20^ given by
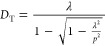
3where λ is the wavelength of illuminating light (193 nm) and *p* is the pitch (center-to-center distance between neighboring holes) of the periodic pattern. The UV laser intensity in the DTL is periodically distributed in the *XY*-plane and spatially spread along the *Z*-axis. In other words, by introducing a variable, displacement of the substrate (with respect to the mask along the *Z*-axis), the optical field can be easily integrated to achieve the spatial distribution of the interference pattern. The DTL process has several advantages over traditional lithography techniques. First, it is insensitive to wafer bowing or uneven sample surfaces, as the 3D exposure method renders patterns with a high depth of focus. Second, it can reproducibly achieve subwavelength resolutions on the wafer scale. Third, the parallel patterning method is extremely fast. The DTL process is described in detail in a previous publication from NanoLund,^20^ in addition to other relevant publications.^22,23^ The details of MACE and DTL are not the focus of this article, but the reader is encouraged to read the previously suggested publications for a broadened understanding of the uses of MACE and DTL.^15,19,22,23^ In this study, we created well-ordered, periodic Si microwires (MWs) with diameters of 200 and 500 nm pitch using a combination of single-exposure displacement Talbot lithography and catalytic etching techniques. The DTL process at NanoLund is developed to fabricate Si MW arrays on a 4-inch wafer, which can be easily transferred to larger wafers based on the geometric size of the phase shift mask. The Si MWs were then coated with mesoporous TiO_2_ and nickel oxide (NiO_*x*_) as a cocatalyst for enhanced aqueous stability and solar seawater catalysis. The effect of periodic Si MWs fabricated using DTL for solar-assisted seawater splitting shows a 65% increase in the net photocurrent density compared to dense Si MWs synthesis using MACE with randomly distributed nanoparticles.^24^

## Results and Discussion

**Figure 1:**
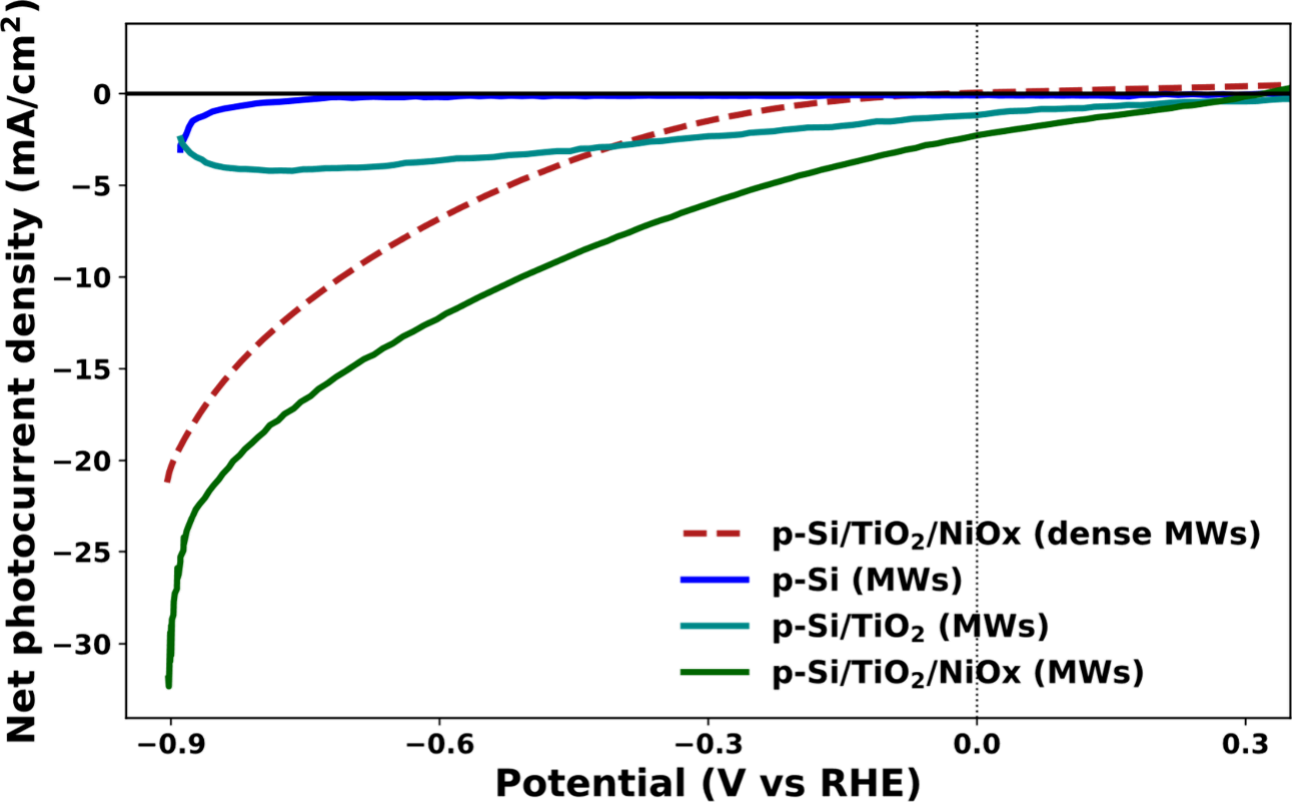
Linear sweep voltammogram showing the net photocurrent density for uncoated, coated, and coated and functionalized periodic Si MWs performed in artificial seawater at pH 8.3. These are compared with the dense Si MWs reported in our previous work.^24^

To estimate the efficiency of these periodic Si MWs as electrodes in solar-assisted seawater splitting, we followed the same procedure as in our previous study for dense, randomly placed Si MWs synthesized using MACE.^24^ In brief, the dense Si MWs were first coated with a thin protective layer of mesoporous TiO_2_, followed by functionalization with NiO_*x*_ as a cocatalyst. The detailed recipe for the synthesis of mesoporous TiO_2_ and NiO_*x*_ precursor was presented in our previous study.^24^ For the characterization as a PEC electrode, the wafer with periodic Si MWs was connected as the working electrode of a typical three-electrode setup with Pt as the counter electrode and Ag/AgCl as a reference electrode. Figure 1 shows the net photocurrent observed during linear sweep voltammetry for the uncoated, coated, and coated and functionalized periodic Si MWs, together with coated and functionalized dense Si MWs^24^ under AM1.5G illumination. Table 1 shows a significantly better net photoelectrochemical performance of our periodic Si MWs compared with the dense Si MWs.^24^ Further, the onset potential, defined as the potential where the net photocurrent is larger than 0.1 mA/cm^2^ in magnitude, is reduced.

**Table 1:** Comparison of Periodic and Randomly Distributed Si MWs^24^ at Selected Potentials

	net photocurrent density and ABPE	
V vs RHE (V)	periodic Si MW @ pH 8.3	dense Si MWs @ pH 8.4
0	–2.3 mA/cm^2^ and 2.8%	0.05 mA/cm^2^ and 0.06%
–0.9	–30.5 mA/cm^2^ and 10.1%	–20.3 mA/cm^2^ and 6.7%
onset potential	0.29 V	–0.12 V

**Figure 2:**
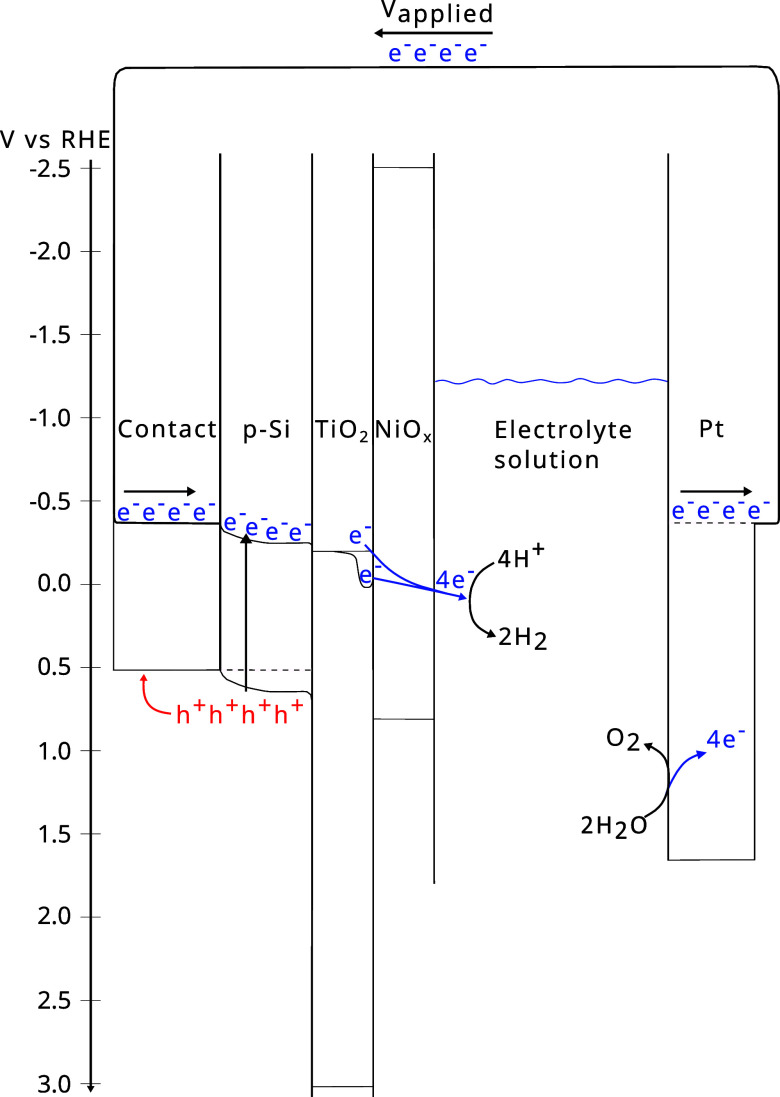
Simplified energy diagram showing the electron transfer from generation in p-Si to reduction of adsorbed H^+^, inspired by refs (10), and (25) with the reported band edges of TiO_2_ from ref (26), NiO_*x*_ from ref (27), and p-Si from ref (25). As suggested in the main text, the TiO_2_ layer introduces oxygen vacancies that can act as electron traps to facilitate electron transfer to adsorbed H^+^. For the sake of clarity, only the electron transfers that contribute to H_2_ generation are shown.

There are several factors that could lead to the increase of 65% at −0.9 V, and a 2.3 mA/cm^2^ enhanced net photocurrent at 0 V. Since the dense Si MWs have been etched randomly, the resulting MWs have asymmetric shapes, dimensions yielding irregular diffusion distances in the order of a few μm through the Si, as seen in the SEMs.^24^ In contrast, the periodic Si MWs are all symmetrical cylinders with diameters near 200 nm, meaning a significantly reduced diffusion distance to the surface, reducing the charge recombination in Si. The symmetrical, periodic shape yields a more uniform loading of mesoporous TiO_2_ and NiO_*x*_, leading to more catalytic sites compared to the dense Si MWs where the silver nanoparticles used in the etching leave many sites inaccessible for functionalization as the SEMs illustrate.^24^ The loading of catalyst and cocatalyst has a significant role for the PEC performance. As previously reported, the layer of mesoporous TiO_2_ enhances PEC performance by introducing oxygen vacancies, as seen in the X-ray diffractogram in the Supporting Information, that act as electron traps localizing the charges and extending the lifetimes for improved charge transfer through the NiO_*x*_ catalyst to solution,^8^ as illustrated in Figure 2. Putting these arguments together, there is a nonuniform loading of the mesoporous TiO_2_ coating and NiO_*x*_ catalyst on the surface of dense Si MWs due to the irregular shapes and diffusion distances that can lead to an extended distance between the points of charge generation and catalyst. This is vastly reduced in the periodic Si MWs, leading to more charges reaching the catalytic sites for hydrogen evolution reaction. We have observed interwire effects that influence the solar light interaction and may further influence the absorption, which we will explore further in the future.

In conclusion, displacement Talbot lithography combined with metal-assisted chemical etching is an efficient tool for the synthesis of highly ordered nanostructures. DTL uses a noncontact transfer of the mask onto the wafer and thus allows exposure free from contamination. We observed a huge improvement of the photocurrent (by 2.3 mA/cm^2^ at 0 V and by 65% at −0.9 V) at pH 8.3 and 1 sun illumination because of this new uniform pattern. We attribute this improvement to more catalytic sites and reduced recombination losses due to the enhanced wire symmetry, leading to more uniform loading of the catalysts, in addition to the reduced diffusion distances.

## Methods

**Figure 3:**
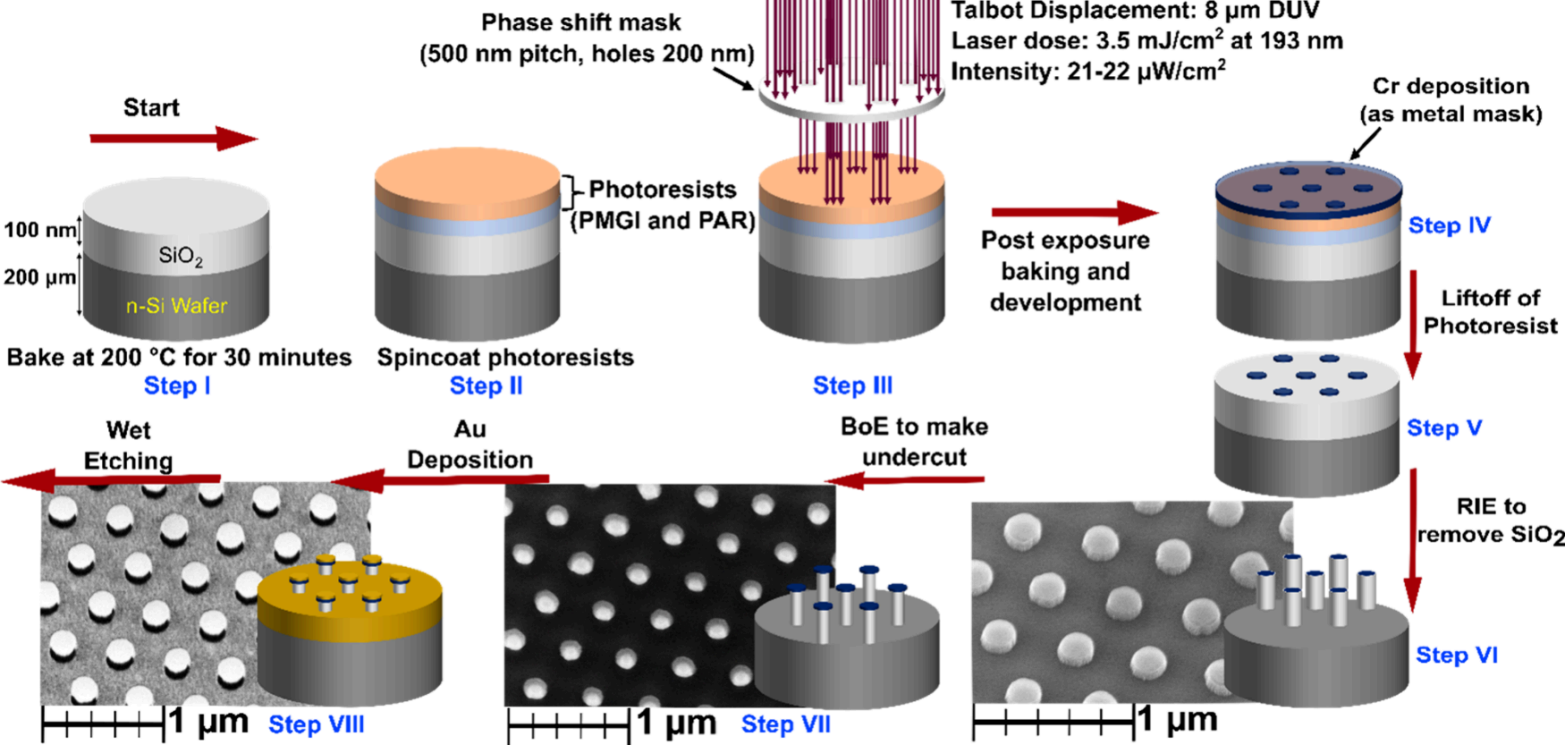
Schematic representation for synthesizing well-ordered, periodic Si microwires using displacement Talbot lithography before metal-assisted chemical etching (not to scale). The SEM images (steps VI–VIII) are taken with a stage tilt of 30°. The bars represent 1 μm with 200 nm increments.

The starting material was a planar 200 μm thick, 2-inch diameter Si (100) wafer with a 111 nm thick thermally induced SiO_2_ layer. First, the wafer was cleaned using acetone, isopropanol, and water under ultrasonication for 10 min each and then dried in a nitrogen stream. The cleaned wafer was baked at 200 °C for 30 min using a hot plate (step I; Figure 3). Directly after the baking, polymethylglutarimide (PMGI-SF3S) (MicroChem Corp, USA) was spin-coated on the Si/SiO_2_ wafer (6000 rpm with 3000 rpm/s acceleration for 45 s) yielding a 70 nm thick layer, followed by 10 min of baking at 200 °C. In the next step, the photoresist (PAR1085S90) (SUMITOMO Chemical Advanced Technologies, SUMIRESIST) was spin-coated using the same spin-coating settings as above, resulting in the same layer thickness, and baked at 90 °C for 1 min (step II; Figure 3). The photoresist-coated Si/SiO_2_ wafer was then exposed to DTL in an Eulitha Phabler R100 with a DUV light source of 193 nm wavelength. For the self-image, a phase shift mask with a hexagonal pattern, 500 nm pitch, and holes of 200 nm diameter was used. The phase shift mask can be varied to generate holes of diameters down to 50–70 nm with a pitch down to 125 nm. From our experience, the lower size limit of the features depends on the pitch and the pattern, and vice versa. Based on the 500 nm pitch, the Talbot displacement was set to 8 μm at a Talbot gap of 80 μm. The pulse energy used for the 193 nm UV-laser was 1.5 mJ at a repetition frequency of 100 Hz at an intensity of 21–23 μW/cm^2^, corresponding to an accumulated dose of 3.5 mJ/cm^2^. After DTL exposure, the sample underwent postexposure baking at 100 °C for 50 s, followed by the development using the ∼2.5% TMAH-based developer MF24A (MicroChem Inc., USA) for 45 s (step III; Figure 3) and then rinsed in deionized water. Onto the patterned Si/SiO_2_ substrate a hard chromium layer (25 nm) was then deposited using electron beam evaporation (step IV; Figure 3) creating a hard mask. This was followed by a liftoff process using the *N*-methylpyrrolidone-based solvent Microposit Remover 1165 (MicroChem Corp, USA) that removes the resist stack together with the chromium on top of the regions not exposed in the DTL. This results in the well-patterned 200 nm Cr circles (500 nm pitch) on the SiO_2_ surface (step V; Figure 3) with up to 10 nm spatial variations. The SiO_2_ on the Si surface was then anisotropically dry-etched using reactive ion etching (RIE) with CHF_3_ plasma and with Sirus T2 Table-top RIE from Trion Technology, except on the Cr sites that act as a protective layer. The operating condition for the RIE was 20 sccm CHF_3_ at 20 mT, 75/64/1 W power (total/forward/reflected) for 420 s.

**Figure 4:**
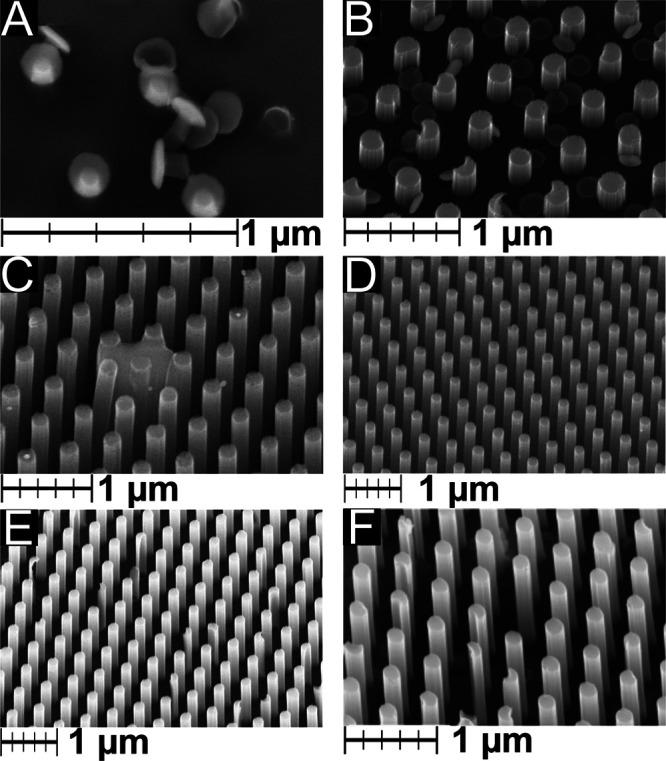
Scanning electron micrographs. (A) SiO_2_/Cr/Au detached from the Si wafer surface. (B) Cr and Au caps on the surface of Si wafer with Si MWs. (C) Si MWs obtained after scotch peeling of SiO_2_/Cr/Au, some of the Au peeled from the Si surface resulting in uneven etching of Si. (D) Si MWs obtained after scotch peeling from a small surface (2 cm^2^). (E) Si MWs obtained after immersing samples from step VIII directly into the etching electrolyte. (F) Si MWs coated with mesoporous TiO_2_. The bars represent 1 μm in 200 nm increments.

This results in SiO_2_ wires at the patterned sites with Cr caps on top (step VI; Figure 3). The SiO_2_ wires are then thinned using buffered oxide etchant (BoE, 10%) for 20 s (step VII; Figure 3), creating an undercut below the Cr caps. Then, 30 nm Au is deposited using electron beam evaporation, creating Cr/Au caps on the wires and a Au film on top of Si on the rest of the wafer. The directionality of the deposition and the undercut results in a gold-free ring around the SiO_2_ wires, creating a pathway for the etching electrolyte to access the metal-Si interface and is essential to form straight wires in the final etching (following step VIII in Figure 3). Before the final etching, however, we needed to consider the Cr/Au caps on the SiO_2_ wires. We found that ultrasonication does help to detach the Cr/Au caps from the SiO_2_, however, it stays on the surface (Figure 4A), leading to uneven etching to yield nonuniform Si MWs (Figure 4B). Scotch tape peeling is another viable alternative to removing SiO_2_/Cr/Au completely. Scotch tape, however, tends to remove some of the gold film from the wafer, which in turn leaves some unetched Si on the wafer surface (Figure 4C). Scotch tape peeling can leave residual on the Si/Au wafer, requiring excessive ultrasonication which in turn can result in the detachment of Si MWs. We found that the most straightforward approach to synthesizing periodic Si MWs is by directly immersing the sample from step VIII (Figure 3) in the etching electrolyte. This may, however, cause the Cr/Au caps to detach from the SiO_2_ , fall onto the silicon, and catalyze uncontrolled etching, resulting in some damaged wires (Figure 4E). The etching electrolyte is made with HF (48%, 23 mL) and H_2_O_2_ (30%, 1 mL) diluted in deionized water (43 mL), and the etching time can be varied to achieve the desired length of microwires (where the etching rate is 200–300 nm/min for the described composition). In this case, the length of the Si MWs was 8.4 μm. The metal-assisted etching mechanism of Si by HF and H_2_O_2_ is well-studied in the literature.^12^

## Supporting Information Available

X-ray diffractograms (Figure S1) highlighting the phases, reflexes, and space groups of p-Si MWs, TiO_2_, and NiO_*x*_ (PDF)
