# Supplementary material for: Synthesis of Well-Ordered Functionalized Silicon Microwires Using Displacement Talbot Lithography for Photocatalysis
Source: ACS Omega. 2024 Apr 25;9(18):20623–8. doi: 10.1021/acsomega.4c03039 (PMC11079887; doi:10.1021/acsomega.4c03039)
Supplement: Supplementary file 1 — ao4c03039_si_001.pdf [file ao4c03039_si_001.pdf]

Supporting information

Synthesis of well-ordered functionalized silicon microwires using Displacement Talbot Lithography for photocatalysis

Axl Eriksson[a,c¥*], Anurag Kawde[a,b,c¥], Lukas Hrachowina[c,d], Sarah R. McKibbin[c,d], Qi Shi[a,c], Magnus T. Borgström[c,d], Thomas Wågberg[e,f], Tönu Pullerits[a,c*], Jens Uhlig[a,b,c*]

[a] Chemical Physics, Department of Chemistry, Lund University, Kemicentrum Naturvetarevägen 16, 223 62 Lund, Sweden.

[b] Lund Institute of Advanced Neutron and X-ray Science, Lund University, Scheelevägen 19, 223 70 Lund, Sweden.

[c] NanoLund, Department of Physics, Lund University, Professorsgatan 1, 223 63 Lund, Sweden.

[d] Solid State Physics, Department of Physics, Lund University, Professorsgatan 1, 223 63 Lund, Sweden.

[e] Department of Physics, Umeå University, Linnaeus väg 20, 907 36 Umeå, Sweden.

[f] Wallenberg Initiative Materials Science for Sustainability, Department of Physics, Umeå University, 901 87 Umeå, Sweden.

X-ray Diffraction Analysis

The X-ray Diffraction (XRD) of p-Si microwires (MWs) and p-Si MWs/TiO_2/NiOx prepared the way described in the Method section of the main text were measured in the single crystal diffraction using a STOE STADI P with Cu K_α irradiation in the Bragg-Brentano geometry.

Figure S1 shows the resulting XRD scans for p-Si MWs (orange), and p-Si MWs/TiO_2/NiOx where the measurement was detuned to suppress the otherwise dominant p-Si contribution to the diffractogram (blue). The XRD analysis reveals monoclinic phases TiO_2 and NiOx, and triclinic $P\bar{1}$ Ti_4O_7 (blue). The TiO_2 has diffraction peaks at 33.7°, 51.5°, 61.5°, and 65.5°, attributed to the (0 0 2), (0 0 3), $(8\ 0\ \bar{1})$, and (5 1 2) reflexes respectively, where the former two belong to C 12/m and the latter two to P 2/m space group. The lattice mismatch of 10 – 20% between TiO_2, Ti_4O_7 and Si phase, and the needle-like texturing of the p-Si MWs causes strain within the coating, which leads to mixed, monoclinic phases of TiO_2. The $P\bar{1}$ triclinic Ti_4O_7 highlights oxygen vacancies in the TiO_2 coating that play a significant role in the photocatalytic efficiency highlighted in the main paper.

Si (100) MWs show a peak at 69.1° that is attributed to the (0 0 4) reflex belonging to the $Fm\bar{3}m$ space group indicating the single crystallinity of the Si (100). In Figure 2 of the main text, the SEMs show no preferential facets which allows higher miller indices such as $(8\ 0\ \bar{1})$ and (5 1 2) reflexes of TiO_2.

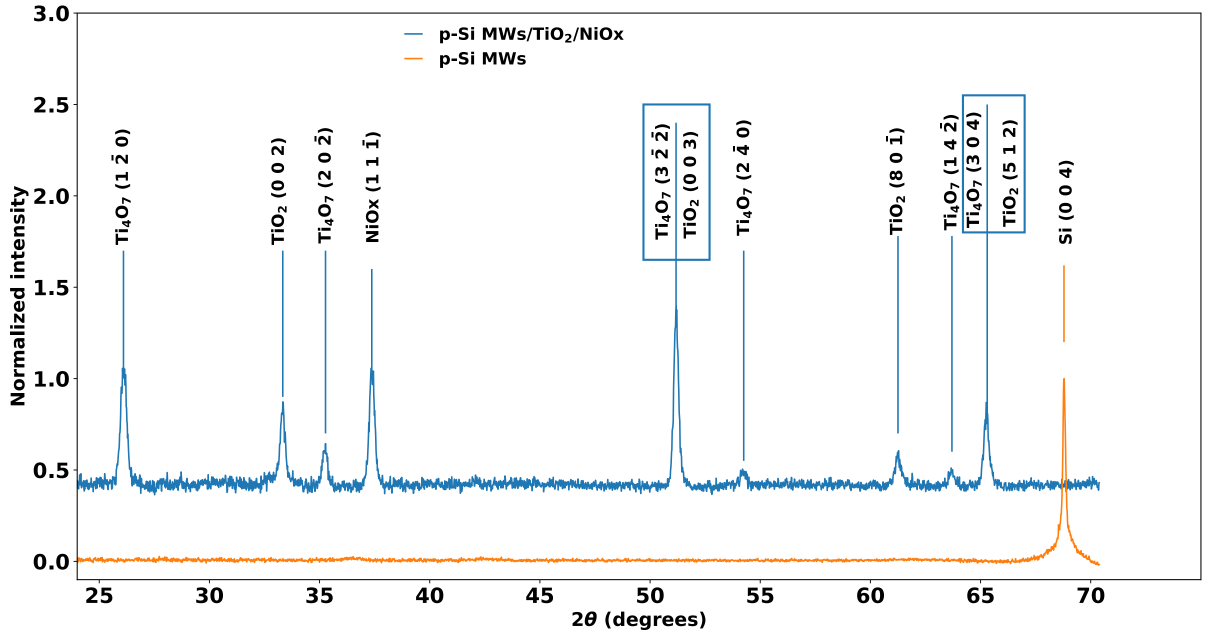

Figure S1. XRD of p-Si MW coated with mesoporous TiO_2 and functionalized with NiOx with suppressed signal from p-Si MW (blue), and uncoated p-Si MWs (orange).
